# Antimicrobial Resistance Determinants and Future Control

**DOI:** 10.3201/eid1106.050167

**Published:** 2005-06

**Authors:** Stephan Harbarth, Matthew H. Samore

**Affiliations:** * University of Geneva Hospitals, Geneva, Switzerland;; † VA Salt Lake City Health Care System, Salt Lake City, Utah, USA

**Keywords:** Antibiotic resistance, infectious diseases, human, Forecasting, multi-drug resistance, Methicillin Resistance, public health surveillance, reducing antimicrobial resistance, Staphylococcus, Epidemiology

## Abstract

At the beginning of the 21st century, antimicrobial resistance is common, has developed against every class of antimicrobial drug, and appears to be spreading into new clinical niches. We describe determinants likely to influence the future epidemiology and health impact of antimicrobial-resistant infections. Understanding these factors will ultimately optimize preventive strategies for an unpredictable future.

"Antibiotic therapy, if indiscriminately used, may turn out to be a medicinal flood that temporarily cleans and heals, but ultimately destroys life itself."

Felix Marti-Ibanez, 1955

For more than 5 decades, the problem of how to contain antimicrobial resistance has preoccupied policy makers and members of the academic community. Nor is this preoccupation surprising, since antimicrobial resistance has become a public health concern throughout the world.

Pessimistic viewpoints about the low chances of success to stop the development of antimicrobial resistance have repeatedly been reported (1). The fundamental predicament is that antimicrobial drugs are a nonrenewable resource. Their duration of benefit and availability appears limited at the biological level, a constraint not seen with therapies for other disease conditions. As pointed out by the commentary in this issue of Emerging Infectious Diseases (2), the emergence of antimicrobial resistance is unavoidable from an evolutionary perspective. Moreover, for most microorganisms, it is unlikely that fitness costs of antimicrobial resistance will reduce their spread and clinical impact, since subsequent evolution commonly results in the amelioration of these costs (3).

This paradigm, which has been framed from a microbiologic perspective and could be summarized in the slogan "antibiotic therapy: use it and lose it," prompts questions about potential interventions that could slow down the dissemination of antimicrobial resistance and reduce its health impact in the next 2 decades. What will influence the demand and use of antimicrobial drugs in the near future? Which obstacles towards more judicious use and decreased transmission may get circumvented? How much will healthcare regulation affect antimicrobial resistance and our ability to control its spread? In short, we need to complement analysis of molecular biology with an examination of other determinants that are likely to influence the future epidemiology and health impact of antimicrobial-resistant, bacterial infections. That is the purpose of this article. For space reasons, we will not discuss the problem of viral, protozoal, or fungal resistance, and the controversial use of antimicrobial drugs in animal growth promotion, but certain analogies may be drawn from the ideas presented here.

## Potential Determinants of the Future Dissemination and Control of Antimicrobial Resistance

Factors that drive uncertainty regarding the future dissemination and control of antimicrobial resistance are numerous and diverse. These determinants can be grouped into 4 categories (Table 1) (4,5). The first group is related to the molecular characteristics of pathogens, such as virulence, transmissibility, and survival fitness, which are issues beyond the scope of this article. Moreover, progress in microbiologic detection and identification of infectious pathogens is likely to influence diagnostic uncertainty and prescribing patterns of antimicrobial drugs. The second group of determinants is linked to prescribers of antimicrobial drugs, physicians, who may change their prescription patterns. Recent data from different parts of the world show promise in this area. The third group is related to characteristics of patient populations and host-related factors. Not only does this include infection rates and case-mix characteristics, but also consumer attitudes and global migration patterns. A fourth group of determinants is linked to macro-level factors related to the healthcare environment. These factors include regulatory policies that may influence use of antimicrobial drugs, infection control practices, technologic development, and drug discovery.

**Table 1:** Potential determinants influencing future dissemination and control of antimicrobial resistance

Dimension	Determinant	Potential control measures and interventions
Pathogen and microbial ecology	Evolution Survival fitness Virulence Commensal flora Laboratory detection and identification	Evolutionary engineering Inhibition of microbial gene expression Antibodies, antipathogenicity drugs, biologic response modifiers Probiotics Improved rapid diagnostic tests
Physician's prescribing practice	Antimicrobial drug usage pattern Diversity of antimicrobial drug prescribing Training and knowledge	Multimodal interventions Decision support tools Academic detailing and educational campaigns
Population characteristics	Migration, travel, and globalization Case mix and host susceptibility to infections Antimicrobial demand and health beliefs Transmission and infection rates	Screening and improved surveillance Immunization; better control of chronic diseases Public information campaigns Hand hygiene and barrier precautions
Politics and healthcare policy	Healthcare policy Promotional activities by industry Technologic development	Change in reimbursement patterns Regulation New prevention and treatment approaches

## Diagnostic Uncertainty and Progress in Laboratory Detection

Diagnostic uncertainty is a key driver of drug misuse and overuse, which can lead to antimicrobial selection pressure and increased rates of resistant microbes (5). The risks associated with untreated microbial infection and the lack of accurate clinical or laboratory prediction methods result in a low threshold for initiating empirical antimicrobial drug therapy, especially if infection could be life-threatening (6).

In the future, diagnosis of microbial infection may be improved at several levels, allowing reduction of antimicrobial selection pressure. First, new diagnostic tests will facilitate initiation or withdrawal of antimicrobial therapy soon after onset of symptoms, especially in the hospital setting. Several new biological markers, such as procalcitonin and soluble triggering receptors expressed on myeloid cells, have been proposed to serve either goal (7,8). Second, molecular diagnostics may increase diagnostic accuracy and enable more prudent antimicrobial drug use in the future. Amplification technology with DNA microarrays and simplified automation opens the potential for rapid testing. Dunne et al. described a scenario in which by the year 2025, sophisticated laboratory platforms with real-time amplifiers will automatically obtain and analyze clinical samples and be able to detect any potentially pathogenic microbe within 30 minutes (9). The threat of bioterrorism may also foster research about rapid molecular diagnostic tests that may be used at the bedside. Third, new diagnostic tools may be available to rapidly distinguish between bacterial and viral infections in the ambulatory setting. Fourth, profound changes will be seen in the techniques used to perform molecular identification and antimicrobial susceptibility testing. In summary, there are several lines of evidence suggesting that a number of molecular, immunologic, and microbial techniques will change the way infectious diseases are diagnosed and reduce diagnostic uncertainty in the next 2 decades (10).

## Prescribing Antimicrobial Agents

To most clinicians, the immediate risk for the patient outweighs the long-term disadvantages of liberal use of antimicrobial drugs. One of the most promising means of reducing antimicrobial selection pressure without impairing patient safety is cessation of antimicrobial drug therapy in patients who do not have a bacterial infection. Great progress has been made within the last 5 years to shorten the duration of treatment with antimicrobial agents (11). Prediction rules designed for the early discontinuation of antimicrobial agents have been validated by prospective trials and will further optimize antimicrobial drug use (12).

Although antimicrobial drug policies and guidelines may not have been of great help in individual decision making, they may have sensitized the medical community to the growing problem of antimicrobial drug overuse and resistance. Consequently, in many industrialized countries, either the number of antimicrobial agent prescriptions or the volume of antimicrobial use has decreased over the last 10 years, especially in the ambulatory setting (Table 2) (13,14). A plateau in worldwide antimicrobial consumption seems to have been reached, leading to a saturated market. As stated recently by representatives of the pharmaceutical industry, "The awareness of the relationship between use and emerging resistance has led to efforts to decrease, even restrict, antibiotic use, and therefore decrease the positive influence of resistance on the market and decrease market potential" (15).

**Table 2:** Countries that have decreased either number of antimicrobial drug prescriptions or total volume of outpatient antimicrobial drugs used within the last 10 years

Continent	Country
Europe	France Belgium Spain Germany United Kingdom Sweden
Asian-Pacific region	South Korea Taiwan Australia
Americas	Canada United States Chile

## Population Characteristics and Technologic Development

Case-mix characteristics and infection rates both inside and outside the healthcare setting will influence antimicrobial drug use and resistance in the future. An increase in immunocompromised patients, the growing life-expectancy, and the susceptibility of older persons to infections could indirectly contribute to greater antimicrobial drug use and dissemination of resistant microbes. Moreover, infectious diseases are influenced by developments in other areas of patient care. New technologies and treatments can create new infectious diseases or eliminate existing ones. For instance, cancer chemotherapy led to new types of susceptible hosts and infectious disease problems, indirectly impelling the dissemination of antimicrobial resistance within hospitals. Key trends in clinical care and biomedical discovery that are likely to influence antimicrobial resistance are the increased use of medical devices and gene therapies, and better management of chronic diseases such as diabetes and cancer. These developments will likely reduce some types of resistance problems and help spawn others.

Global threats such as the next influenza pandemic may also affect prescribing of antimicrobial drugs by reversing the trend of decreasing antimicrobial drug consumption (16). Conversely, climate change may lead to a decrease in respiratory tract infections and antimicrobial drug use in the winter months (17).

## Travel and Globalization

Globalization and migration into mega-cities has led to new possibilities of cross-transmission of antimicrobial resistance (1). Recent events such as the terrorist attack in Bali, the war in Iraq, and the tsunami in Southeast Asia have led to the transfer of patients infected with panresistant gram-negative bacteria such as *Acinetobacter* spp. to other parts of the world, causing outbreaks and public health concerns (18). Within the next 2 decades, global mixing, increased population density, and decreased travel times will facilitate the spread of a variety of antimicrobial-resistant pathogens such as fluoroquinolone-resistant pneumococci and enteric microbes.

Since antimicrobial resistance is influenced by international travel and globalization, resistance may, in turn, affect how nations respond to each other. Especially as surveillance systems improve in quality, international pressure may be applied to induce change in countries where antimicrobial agents are abused or where infection control policies are lax. The situation in antimicrobial resistance might become comparable to that which exists for other infectious problems such as mad cow disease: economic pressure may contribute to compliance and uniformity in control measures. Nevertheless, approaches to control the global spread of resistance will remain difficult to implement and will require intensive surveillance and screening efforts.

## Health Beliefs and Antimicrobial Drug Demand

Although the interplay between health beliefs and demand of antimicrobial drugs is widely recognized, few, if any, systematic studies exist about the future influence of the cultural setting on antimicrobial drug use and related resistance rates (19). Social constraints and cultural views of infectious conditions that require antimicrobial treatment exert a strong influence on their use, particularly for community-acquired pathogens.

**Figure 1:**
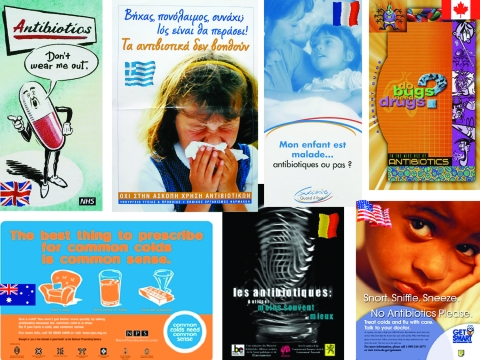
Posters from nationwide educational campaigns against misuse of antimicrobial drugs.

Several countries have recently taken the bold step of launching national campaigns to educate physicians and patients about antimicrobial misuse and the threat of resistance (Figure 1). These campaigns show promise in changing attitudes and behavior, among both the public and healthcare professionals (20). If repeated regularly, the campaigns are likely to reduce inappropriate patient requests for antimicrobial agents, which in conjunction with physician education models may reduce inappropriate antimicrobial prescription practices (21). Ultimately, they may slow the dissemination of certain antimicrobial-resistant pathogens (5). For nstance, in several countries, such as France and Spain, which use a great amount of antimicrobial agents, a decrease in pneumococcal resistance rates among invasive isolates has been noted recently. This coincides with a decrease in antimicrobial drug use after nationwide campaigns and the introduction of a conjugate pneumococcal vaccine (22). Nevertheless, uncertainty persists about possible negative outcomes and countermeasures taken by the pharmaceutical industry to oppose these campaigns.

## Vaccinology

Modern vaccinology (the development of new vaccines) is likely to contribute to the decreased transmission and impact of antimicrobial-resistant bacteria in the near future (23). More so than antimicrobial agents, vaccines have the potential to durably control infectious agents by blocking their ability to disseminate within a population. This expectation can be illustrated by the example of the new pneumococcal conjugate vaccine. Based on encouraging results from countries with high prevalence of pneumococcal resistance such as Israel, France, Spain, and the United States, this vaccine will likely reduce the incidence of invasive disease due to resistant pneumococci (22,24). Further progress in pneumococcal vaccine development can be expected from conjugate vaccines that include more than 7 serotypes (25). Yet uncertainty remains regarding serotype replacement and the emergence of resistance in nonvaccine serotypes (25).

Other vaccines to prevent invasive, antimicrobial-resistant infections will be launched within the next 20 years (23). Potential candidates are vaccines against multidrug-resistant staphylococci and enterococci, but clinical studies need to confirm promising preliminary results.

## Infection Control in the Healthcare Setting

While the intense selective pressure of antimicrobial drug use has been an important factor in the emergence of resistance, the inconsistent application of infection control guidelines by hospital personnel largely accounts for the dissemination of resistance in the healthcare setting. Infection control measures to limit the spread of antimicrobial resistance are being increasingly well defined. Despite the increase in the prevalence of resistance of several important pathogens, there has been some success in controlling its clinical impact. Several countries have recently reported a stabilization or decrease in infection rates due to multidrug-resistant *Staphylococcus aureus* (26).

The next 20 years will see an increase in infection control research and interventions to improve patient safety. Hand hygiene with alcohol-based hand rubs has been shown to decrease the transmission of resistant organisms (27). A campaign sponsored by the World Health Organization in 2005 is promoting its practice throughout the world. Early screening and isolation of patients carrying resistant organisms also appear to decrease the spread of resistant microorganisms and may be more widely implemented (28). Some experts have suggested that multimodal approaches that use a combination of different measures (for example, aggressive infection control with active surveillance cultures, hand hygiene, and possibly antimicrobial control) will effectively slow down and even halt the increasing trends of healthcare-associated antimicrobial resistance (29).

## Healthcare Regulation

Antimicrobial use is affected by reimbursement policies, financial incentives, and healthcare regulation (19). Forecasting the political and regulatory development in this area presents a major challenge. There is always a short-term lack of predictability with regard to political decision-making after unexpected epidemiologic situations, such as the bioterrorist attacks in 2001 and severe acute respiratory syndrome in 2003, which quickly influenced perceived medical needs (30).

Looking at the future impact of healthcare regulation, many believe that political measures to control antimicrobial drug use have only a negligible short-term effect (1,31). We argue, however, that healthcare regulation will powerfully influence antimicrobial drug use in the future. To underline this hypothesis, we give 3 examples from different continents.

## Interdiction of Over-the-Counter Sales of Antimicrobial Agents in Chile

**Figure 2:**
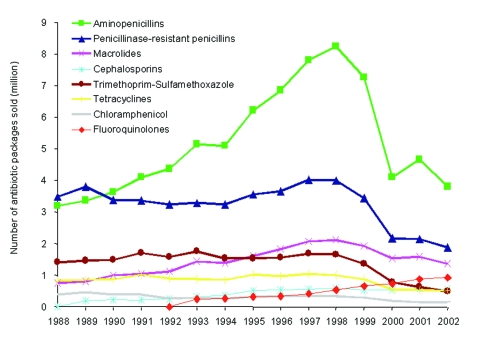
Number of antimicrobial drug packages sold in the outpatient setting in Chile, 1988–2002. Package is the term used to show sales figures of antimicrobial drugs from wholesalers or pharmacies. It is also used to calculate the number of daily defined doses for each marketed antimicrobial drug. Data are from Bavestrello et al (32). Unpublished data from 2001 and 2002 were provided by A. Cabello Munoz and L. Bavestrello (Viña del Mar, Chile).

Self-medication is an important driver of antimicrobial overuse in low- and middle-income countries. Therefore since 1999, the Chilean Ministry of Health has strictly enforced existing laws, which restricted purchase of antimicrobial agents without a medical prescription. These regulatory measures had a sustained impact on antimicrobial use in the outpatient setting: sales of orally used antimicrobial agents decreased by 43% from US $45.8 million in 1998 to US $26.1 million in 2002 (Figure 2) (32).

## Restriction of Perioperative Antimicrobial Prophylaxis in Belgium

Inadequate and prolonged perioperative antimicrobial prophylaxis increases resistance to antimicrobial drugs (33). In 1997, a Royal Decree in Belgium limited reimbursement of antimicrobial drug prophylaxis to specific agents and a 24-hour period after surgery (34). Moreover, a fixed fee for antimicrobial costs was attributed to each type of intervention. As shown in Table 3, this regulatory restriction had a sustained effect on the use of antimicrobial prophylaxis in Belgium (34).

**Table 3:** Proportions of appropriate perioperative antimicrobial drug prophylaxis in Belgian hospitals after change in the reimbursement system*

	1986 (%)	1999 (%)
Correct timing	53	70
Correct indication	92	97
Duration >48 h	50	8
Choice of agent		
First-generation cephalosporin	28	66
Second-generation cephalosporin	17	29

*Source: Goossens et al (34).

## Separation of Dispensing and Prescribing in South Korea

In Europe and North America, separation of antimicrobial prescribing and dispensing is a well-established system. In contrast, in many Asian countries, healthcare providers earn a significant proportion of their income from dispensing drugs, especially broad-spectrum antimicrobial agents (35). Consequently, physicians have traditionally compensated for relatively low medical service revenue by prescribing a high volume of antimicrobial agents. In 2000, against the strong opposition of physicians and the pharmaceutical industry, a new Korean government policy prohibited physicians from dispensing drugs and pharmacists from prescribing drugs (36). This new policy decreased overall prescribing of antimicrobial agents and selectively reduced inappropriate prescribing of them for patients with viral infections (36).

## Future Directions

The uncertainty evolving around micro- and macro-level determinants influencing antimicrobial resistance makes long-term prediction challenging. Although simulation studies may provide guidance about short-term trends (37), long-term predictions about the future of antimicrobial resistance are fraught with difficulties, as shown by a look back in history. When the antimicrobial drug era began, scientists were impressed by the milestones of antimicrobial agent discovery and issued predictions about the future of antimicrobial resistance that seem overly optimistic today (38). For instance, in 1952, a famous French microbiologist anticipated pneumococci, gonococci, and meningococci would not change their antimicrobial susceptibility profile in the future ("Pour une espèce qui au départ était entièrement sensible…, l'espèce sera toujours aussi sensible. C'est le cas des germes très sensibles à la pénicilline: gonocoques, pneumocoques, méningocoques") (39). Yet exactly 40 years later, we were rapidly progressing towards a "post-antimicrobial era" in which doctors may become helpless against even common infections (40).

In the last part of this article, we contemplate the possible status of antimicrobial resistance in 2025. Although the direction of a few major trends seems relatively easy, other factors that drive uncertainty present tremendous forecasting challenges. Therefore, we have developed 2 alternative scenarios about the future dissemination and control of antimicrobial resistance. These were extrapolated from the key determinants discussed earlier. The informed reader of 2025 may apologize for our lack of imagination.

## What Will Be the Status of Antimicrobial Resistance in 20 Years?

The Bright Scenario

*We will observe a change in prescribing habits and attitudes towards outpatient antimicrobial use, especially for respiratory infections.* Policies and behavior change interventions contribute to a massive change in social norms around antimicrobial drug use, similar to what has happened with tobacco control. Intensive educational campaigns, aimed at optimizing antimicrobial drug use, combined with immunization programs for infants and children will lead to reduced spread and clinical impact of antimicrobial-resistant pneumococci.

*Tools from information technology and progress in microbiology will reduce diagnostic uncertainty and improve antimicrobial dosing, selection, and treatment duration.* Use of antimicrobial agents will, therefore, continue to decrease, not only in the outpatient setting, but also in the inpatient setting.

*New therapies will be developed based on probiotic principles.* Technologic advances will enhance the identification and characterization of the vast microbial diversity colonizing the human body (commensals and pathogens), which may lead to new probiotic strategies to prevent infections and reduce antimicrobial selection pressure.

*Data sharing and increased international cooperation will lead to consistent control measures across different continents.* Asian countries, users of large amounts of antimicrobial agents and important drivers of resistance until recently, will change paradigms and introduce modern infection control concepts and public health policies that will decrease overuse of antimicrobial agents.

*Antimicrobial resistance among important pathogens will be slowly reversible.* Trends in antimicrobial resistance follow an S-shaped curve with a quick ascent, a plateau and, sometimes, a slow decline. Antimicrobial resistance in high prevalence countries will be slowly reduced, especially for several gram-positive microorganisms (1).

*Antimicrobial resistance will not have a major impact on life expectancy in the industrialized world.* Deaths from panresistant infections without any treatment option will remain rare complications in high-income countries, since new antimicrobial agents and better use of currently available antimicrobial drugs will become standard policy.

### The Dark Scenario

*New resistance mechanisms will emerge and disseminate.* Multiresistant group A streptococci will render penicillin and macrolides useless in the treatment of pharyngitis. *Salmonella* spp. infections can no longer be treated with advanced cephalosporins, fluorquinolones, or carbapenems.

*We will observe raising resistance rates for most pathogens.* Multiresistant *Acinetobacter* spp., enterococci, and staphylococci will cause substantial illness and increased treatment costs in those parts of the world that have not installed stringent control measures. Healthcare-associated infections due to vancomycin-resistant enterococci will become endemic in many countries.

*Antibiotic-resistant S. aureus will become a massive public health problem.* The scope of staphylococcal antimicrobial resistance will extend not only to new antimicrobial agents, but also to more settings. Although hospitals were once the sole province of methicillin-resistant *S. aureus* (MRSA), more and more community outbreaks of MRSA will occur in those persons who lack traditional risk factors for carriage of MRSA. The prevalence of MRSA in the US community will reach 25% within the next decade, with rates 3 times as high in hospitals (41).

Technological development will not fulfill its promise. *No new antimicrobial classes or treatment strategies* have been developed for gram-negative bacteria, and vaccines have not been widely effective. Serotype replacement in pneumococci allowed that organism to escape control. Fluoroquinolones are no longer effective against a wide array of infections and have not been replaced by any new class of orally available antimicrobial agents. New antimicrobial drugs with novel mechanisms of action (e.g., bacteriophages) have failed in large phase III trials.

*Anthrax and pandemic influenza threats have led to mass prophylaxis, with disastrous consequences in terms of resistance*. Several disasters and pandemics will increase the use of antimicrobial drugs on a global scale, leading to emergence and dissemination of resistance.

*A continuing flood of consensus conference statements, position papers, and surveillance network reports will be issued about the problem of antibiotic resistance, without any measurable and sustained effect on containment.* Healthcare policy will not introduce stringent control measures because of a lack of precise estimates of the public health impact of antimicrobial resistance and the priority of other more pressing infectious disease problems such as HIV, tuberculosis, and malaria.

### Conclusion

The high levels of uncertainty and complexity regarding antimicrobial resistance mandate that we build the capabilities to prepare not only for 1 specific future (following the pessimistic viewpoint of antimicrobial therapy: use it and lose it), but also across a range of alternative scenarios that may be less pessimistic.

Whether the current epidemic of antimicrobial resistance is sustainable or will succumb to current efforts to limit its spread will be decided by an interaction of factors related to microorganisms, host, use patterns of antimicrobial drugs, and the impact of infection control measures and technologic development (5). We hope that adding infection control and prudent use of antimicrobial agents to new drug development will avert the realization of pessimistic predictions about the future of antimicrobial resistance.
